# Clinical Validation of a New Tinnitus Assessment Technology

**DOI:** 10.3389/fneur.2017.00038

**Published:** 2017-02-21

**Authors:** Sylvie Hébert, Philippe Fournier

**Affiliations:** ^1^Faculty of Medicine, School of Speech Pathology and Audiology, Université de Montréal, Montréal, QC, Canada; ^2^BRAMS – International Laboratory for Research on Brain, Music, and Sound, Université de Montréal, Montréal, QC, Canada

**Keywords:** tinnitus, psychoacoustic test tool, loudness, pitch, hearing loss

## Abstract

Current clinical assessment of tinnitus relies mainly on self-report. Psychoacoustic assessment of tinnitus pitch and loudness are recommended but methods yield variable results. Herein, we investigated the proposition that a previously validated fixed laboratory-based method (Touchscreen) and a newly developed clinically relevant portable prototype (Stand-alone) yield comparable results in the assessment of psychoacoustic tinnitus pitch and loudness. Participants with tinnitus [*N* = 15, 7 with normal hearing and 8 with hearing loss (HL)] and participants simulating tinnitus (simulators, *N* = 15) were instructed to rate the likeness of pure tones (250—16 kHz) to their tinnitus pitch and match their loudness using both methods presented in a counterbalanced order. Results indicate that simulators rated their “tinnitus” at lower frequencies and at louder levels (~10 dB) compared to tinnitus participants. Tinnitus subgroups (with vs. without HL) differed in their predominant tinnitus pitch (i.e., lower in the tinnitus with HL subgroups), but not in their loudness matching in decibel SL. Loudness at the predominant pitch was identified as a factor yielding significant sensitivity and specificity in discriminating between the two groups of participants. Importantly, despite differences in the devices’ physical presentations, likeness and loudness ratings were globally consistent between the two methods and, moreover, highly reproducible from one method to the other in both groups. All in all, both methods yielded robust tinnitus data in less than 12 min, with the Stand-alone having the advantage of not being dependent of learning effects, being user-friendly, and being adapted to the audiogram of each patient to further reduce testing time.

## Introduction

Tinnitus is a common health condition that affects from 11.9 to 30.3% of the general population and its prevalence increases with increasing age (1, 2). Tinnitus can interfere with daily life and is associated with significant psychological distress, anxiety, and other health-related issues (1). Individuals with tinnitus seeking clinical services are at a clear disadvantage compared to others because there are no established guidelines for the clinical assessment and intervention for tinnitus. At the basis of clinical service is assessment, which confirms diagnosis, as well as determines and monitors intervention, and yet, current clinical assessments of tinnitus mostly rely on patients’ self-report.

Tinnitus can be characterized by its psychoacoustic properties (pitch and loudness), which pertain to the auditory domain, and by its associated distress, which pertains to the psychological domain. Some clinical settings assess psychoacoustic measurements of pitch and loudness. Classical forced-choice paradigm and the method of adjustment are the preferred methods used (3). However, the procedure is passive, with clinicians controlling the stimulus parameters presented to the patient (3). These techniques are usually mastered by highly skilled clinicians and yet do not provide stable measurements of the tinnitus percept within a session or between sessions over time (3). This lack of reliability compromises tinnitus assessments such that clinical trials often have to rely on visual analog scales or tinnitus questionnaires as main treatment outcomes (4, 5). These outcomes, however, are highly unsatisfactory because some therapeutic interventions (e.g., transcranial magnetic stimulation of the auditory cortex, deep brain stimulation, noise-notched music) are targeted primarily at decreasing the psychoacoustic loudness of tinnitus, which in turn would supposedly decrease the associated psychological distress. However, the precise relationship between loudness and distress is unknown and usually statistical correlations are not very high (6). Therefore, the best tinnitus assessment should include reliable measures of both of these aspects.

Recently, an active method allowing the patient to control parameters using a Touchscreen has shown good test–retest reliability for tinnitus predominant pitch and loudness matching over several months (6, 7). Moreover, psychoacoustic tinnitus loudness matches were higher for simulators than for participants with tinnitus and was a better predictor for specificity (e.g., correctly detecting simulators) than predominant pitch (4). A similar method specifically designed for clinical settings could be very useful for health-care professionals to assess the progression of the tinnitus over several months in individual patients and assess treatment’ efficacy with confidence. High levels of sensitivity and specificity of the method are interesting features that would be valuable in medicolegal cases of tinnitus. For these purposes, a new Stand-alone prototype (hereafter, Stand-alone) was developed from the first laboratory device (hereafter, Touchscreen), in order to capture the main tinnitus characteristics—likeness and loudness ratings—but in a format that is more suited for clinical purposes.

The aim of the present study was, therefore, to compare performance of the two methods, including participants simulating having tinnitus. As previously reported, the tinnitus likeness and most importantly the loudness should be significantly different between tinnitus participants and simulators for both methods. Likewise, loudness should be a better predictor of tinnitus presence compared to tinnitus predominant pitch, again for both methods.

As time efficiency is a major issue for clinicians, testing time for the two devices was assessed and compared. We predicted that the modification made to the Stand-alone device should provide a more efficient testing time than the Touchscreen method for patients with hearing loss (HL). Finally, the effect of presenting two pure tones instead of the standard three pure tones was assessed, as it could also potentially reduce testing time.

A second objective was to compare two tinnitus subtypes. In particular, tinnitus with and without HL has been shown to engage two different types of brain structures (8, 9). In addition, differences in tinnitus spectrum have been noted across studies between tinnitus patients with and without HL mostly in the very high frequency region (6, 10). From a clinical standpoint, these two groups are also very different in term of assessment and therapeutic approach. For instance, hearing assessment for tinnitus patients with HL is usually longer than for patients without HL, and thus, any saved time in the assessment of tinnitus would be a valuable asset for a clinical device. In that respect, tinnitus subgroups were further examined on the basis of this criterion.

## Materials and Methods

### Participants

Two groups of participants who had not participated in our previous studies using the Touchscreen method were recruited through word of mouth or advertisements in local newspapers. Tinnitus participants had to have chronic bilateral tinnitus, not have complete deafness in one or both ears, and be in good health. Health was further verified by questions on possible diseases (e.g., neurological disease), conditions (e.g., uncontrolled hypertension), and medication. This group consisted of 15 adults (9M, 6F) with a mean age of 45 years (min: 23; max: 69). Simulator participants had to have had a previous experience of transient tinnitus (less than a day) more than 3 months before the experiment so that they could rely on this past experience to fake tinnitus. Most participants reported having previous experience of transient tinnitus after loud sound exposure such as a music concert, a very frequent phenomenon (10). This group consisted of 15 adults (7M, 8F) with a mean age of 32 years (min: 21; max: 62). They were instructed to simulate this sound perception with the intention of convincing the experimenter that they had tinnitus. None of the participants reported otologic condition other than HL and none of the participants were smokers.

### Tinnitus Subtypes

The normal hearing (NH) Tinnitus subgroup (*N* = 7) had hearing thresholds ≤25 dB hearing loss (HL) at all frequencies from 250 to 8 kHz in both ears. The HL tinnitus subgroup (*N* = 8) had hearing thresholds >25 dB HL at least at one frequency between 250 and 8 kHz in either ear.

### Hearing Assessment

An otoscopy was performed in order to rule out earwax compaction and outer ear pathology. Standard hearing detection thresholds were assessed in each ear monaurally from 0.25 to 8 kHz in half-octave steps by a clinical audiologist using the standard modified Hughson–Westlake up–down procedure (5) with an AC-40 clinical audiometer and ER-3A insert earphones in a sound-proof booth (ANSI S3.6-2004 standard norms). Very-high frequency thresholds (9–16 kHz) were also assessed monaurally in each ear using Sennheiser HDA-200 supra-aural headphones.

### Tinnitus Matching Method

**Figure 1:**
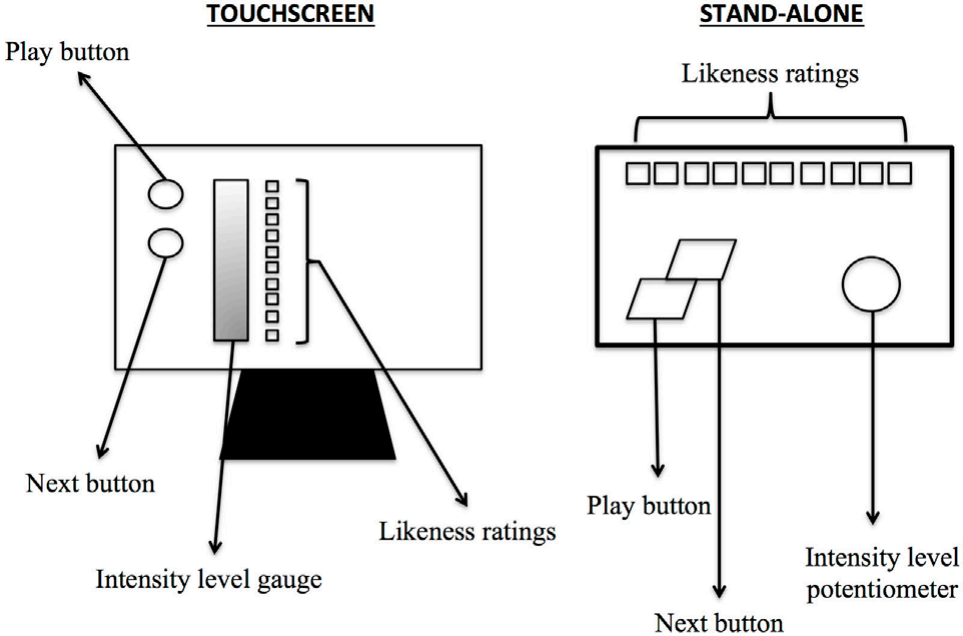
**Schematization of the Touchscreen and the Stand-alone devices used in the experiment**.

Tinnitus assessment was run with both the Touchscreen and the Stand-alone in each participant in a counterbalanced order. Both methods used 3-s pure tones ranging from 0.25 to 16 kHz presented three times in a pseudorandom order such that no two identical frequencies were presented in a row. Participants were first asked to rate the likeness of the tone to their tinnitus pitch on a Likert-type scale in which 0 = “does not match my tinnitus at all” and 10 = “perfectly matches my tinnitus.” During the same trial, they had to match the loudness of the tone—that is, the sound level at which that specific frequency contributed to their tinnitus—by moving a visual gauge (Touchscreen) or a potentiometer (Stand-alone) that increased and decreased the sound level by 1 dB steps. Participants were allowed to play each pure tone as many times as needed for both methods. The sound was presented binaurally using closed DT 770 Pro/250 dynamic headphones (Beyerdynamic, Heilbronn, Germany) for the Touchscreen method and HDA-300 Sennheiser (Sennheiser electronic GmbH & Co. KG, Germany) headphones for the Stand-alone device. The main difference between the two methods (see Figure 1) is that the Stand-alone uses standard buttons for playing sounds and rating likeness and a potentiometer for loudness matching. The design of the Stand-alone maximizes ergonomics and comfort to improve its use by older patients.

In addition, in order to make the method more time-efficient, especially for participants with some degree of HL, an additional step was implemented in the Stand-alone method. The audiogram of each participant was taken into account such that (1) frequencies for which a threshold could not be found (i.e., higher than the limits of the audiometer) were not presented and (2) levels of the frequencies presented were always beyond threshold. This information was added to the device just before the task began.

### Calibration

Headphones were calibrated before each session with a SoundPro SE/DL sound level meter using a QE-4170 microphone model (Quest Technologies, Oconomowoc, WI, USA) and an EC-9A 2cc ear coupler (Quest Electronics, Oconomowoc, WI, USA).

### Procedure

All participants were first assessed with the hearing evaluation. Then, they were tested with one of the two matching methods (Touchscreen or Stand-alone) in a counterbalanced order. They were next tested with the other method, both taking place in the same sound attenuated room. Participants were asked to provide repeatable tinnitus matching responses to the best of their ability. Simulators were instructed to use their past experience of tinnitus to convince the experimenter that they had tinnitus but were not instructed to use any particular method to provide consistent responses during matching. The time required to perform the tinnitus-matching task for each device was assessed using a conventional chronometer. The experimenter started the chronometer as soon as the participant press the « play » button for the first time, that is, after the instruction was given for each device individually. The study was approved by the ethics committee of Université de Montréal and was conducted with the understanding and written consent of each participant.

### Analyses

In all analyses below, the two groups (Tinnitus vs. Simulators) were first compared, and in a second step, the Tinnitus subgroups (NH vs. HL) were compared. Hearing thresholds were analyzed with a 2 × (2 × 16) ANOVA with Group (first, Tinnitus vs. Simulators; second, Tinnitus NH vs. HL) as a between-subject factors and ear (right, left) and frequency (250–16 kHz) as within-subject factors. Similar analyses were run on likeness ratings and loudness matching but with Methods (Touchscreen vs. Stand-alone) rather than ear as a within-subject factor. Product-to-moment Pearson correlations and paired-sample *T*-tests were run on these data to compare the two methods. The first and second predominant pitches and their corresponding loudness were extracted from the tinnitus spectrum of each participant and compared between groups and subgroups as described above.

Sensitivity and specificity analyses were run for each method (Touchscreen, Stand-alone) using logistic regressions taking group as the dependent factor putting the first predominant pitch and its corresponding loudness match as independent predictors.

To investigate potential learning effects in using devices, testing time was analyzed using a 2 × (2 × 2) ANOVA with Group [(1) Tinnitus vs. Simulators; (2) Tinnitus NH vs. HL] as between-subject factors and order of presentation (Touchscreen first vs. Stand-alone first) and Method (Touchscreen vs. Stand-alone) as within-subject factors. Finally, comparisons were made between using all three instances of pure tones and the first two instances only.

## Results

### Hearing Thresholds

**Figure 2:**
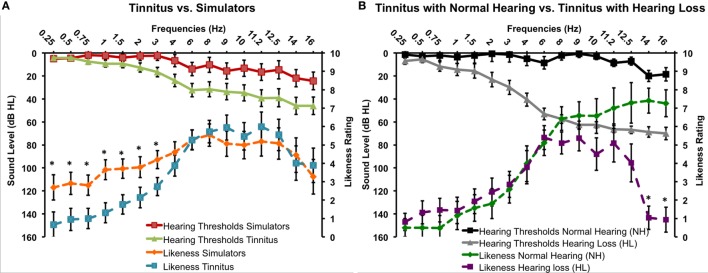
**Best hearing thresholds and tinnitus likeness ratings for the Tinnitus and Simulator groups (A) and the two Tinnitus subgroups with normal hearing and with hearing loss (B) (SEM)**.

Hearing thresholds of all 16 frequencies did not differ across ears (all *p*s >0.05) except at 12.5 kHz (*p* = 0.001) and, therefore, were averaged. When considering two groups (Tinnitus, Simulators), the two-way interaction between frequency and group was significant, *F*(15, 420) = 3.32, *p* < 0.001. Thresholds for the Tinnitus group were higher from 3 to 14 kHz, all *p*s <0.05 and only marginally higher at 16 kHz, *p* = 0.07. When considering only the tinnitus subgroups NH vs. HL, the same pattern emerged, *F*(15, 195) = 23.59, *p* < 0.001. Thresholds for the HL subgroup were higher from 2 to 16 kHz, all *p*s <0.04. Figure 2 displays the best thresholds for the three groups for Tinnitus and Simulator group (Figure 2A) and Tinnitus subgroups NH and HL (Figure 2B).

### Tinnitus Pitch Matching

On likeness ratings, there was no significant main effect of Methods or any interaction involving this factor (all *p*s >0.05), suggesting that the two methods work similarly (see Table 1). Paired-sample *T*-tests revealed only one significant difference at 9 kHz with a seven mean difference in the ratings between the two methods (see Table 1). Overall, correlations between the two methods were very high (mean *r* = 0.74, min: 0.53, max: 0.95). Figure 2A shows the likeness ratings for the Tinnitus and Simulator groups with merged Methods. There was a two-way interaction between Group and Frequency, *F*(15, 420) = 1.70, *p* < 0.05. Simulators’ ratings were higher compared to Tinnitus’ for frequencies 250–3 kHz irrespective of Methods, all *p*s <0.05.

**Table 1:** **Mean differences and product-to-moment Pearson correlations between the two methods (Touchscreen vs. Stand-alone) and the two groups (Simulators and Tinnitus groups merged) for likeness ratings and loudness matching at each frequency**.

	Likeness ratings				Loudness matching			
Frequency (kHz)	Mean difference	*p*-Value	*r*	*p*-Value	Mean difference (dB)	*p*-Value	*r*	*p*-Value
0.25	0.1	0.70	0.95	<0.001	−0.4	0.76	0.88	<0.001
0.5	0.2	0.59	0.76	<0.001	−0.4	0.88	0.78	<0.001
0.75	−0.2	0.33	0.85	<0.001	−2.3	0.49	0.70	<0.001
1	0.3	0.33	0.79	<0.001	−1.9	0.56	0.71	<0.001
1.5	−0.4	0.27	0.66	<0.001	−5.0	0.15	0.67	<0.001
2	−0.3	0.43	0.66	<0.001	**−8.4**	**0.05**	0.56	0.001
3	−0.4	0.21	0.64	<0.001	**−14.0**	**<0.001**	0.74	<0.001
4	−0.4	0.37	0.62	<0.001	**−7.5**	**0.05**	0.69	<0.001
6	−0.3	0.46	0.53	0.003	−5.3	0.07	0.68	<0.001
8	0.2	0.64	0.65	<0.001	5.9	0.07	0.65	<0.001
9	**0.7**	**0.04**	0.81	<0.001	**9.9**	**0.01**	0.56	0.001
10	0.1	0.87	0.81	<0.001	4.4	0.11	0.73	<0.001
11.2	0.1	0.71	0.87	<0.001	1.0	0.72	0.61	<0.001
12.5	0.4	0.37	0.76	<0.001	−7.7	0.07	0.44	0.014
14	**1.0**	**0.04**	0.76	<0.001	1.6	0.55	0.67	<0.001
16	0.9	0.09	0.76	<0.001	0.0	1.00	0.26	0.164
Grand mean	0.1		0.74		−1.9		0.65	

*Significant differences between the two methods are in bold*.

For the Tinnitus subgroups, there was no effect of Methods or interaction involving this factor (all *p*s >0.05). Figure 2B shows the likeness ratings for the two subgroups NH and HL tinnitus. There was a significant interaction between frequency and subgroups, *F*(15, 195) = 5.16, *p* < 0.001. Ratings were higher for frequencies 14 and 16 kHz for the NH subgroup (*p* < 0.001) and marginally significant for the 12.5 kHz (*p* = 0.05). None of the other frequency differed (all *p*s >0.14). There was also a main effect of subgroup, *F*(1, 13) = 7.02, *p* = 0.02, with NH tinnitus subgroup rating their tinnitus with higher scores than the HL subgroup (means of 4 and 3, respectively).

When considering the first and the second predominant pitch, that is, the frequencies with the first and the second highest ratings in each individual participant, the Tinnitus group did not differ from the Simulator group (see Table 2). Interestingly, the Tinnitus subgroups differed from one another in terms of the first predominant pitch, with a higher predominant pitch for the NH subgroup compared to the HL subgroup (see Table 2).

**Table 2:** **Comparisons between the first and second predominant pitches of the tinnitus spectrum (in kHz) and their corresponding loudness (in dB SL) for the two methods**.

	Tinnitus	Simulators	*p-*Value	Tinnitus with normal hearing	Tinnitus with hearing loss	*p-*Value
**Touchscreen**						
First predominant pitch (kHz)	11.37	8.98	n.s.	14.64	8.5	**=0.001**
Second predominant pitch (kHz)	9.06	9.86	n.s.	11.03	8.84	n.s.
Loudness predominant pitch (SL)	−1.9	20	**=0.015**	−10	5.2	n.s.
Loudness second predominant pitch (SL)	6.1	22	**=0.046**	6.3	5.9	n.s.
**Stand-alone**						
First predominant pitch (kHz)	11.09	8.39	n.s.	14	8.55	**=0.02**
Second predominant pitch (kHz)	10.01	7.06	n.s.	12.21	8.08	**=0.04**
Loudness predominant pitch (SL)	10.8	24.92	**=0.02**	5.75	15.2	n.s.
Loudness second predominant pitch (SL)	9.18	27.48	**=0.002**	5.7	12.23	n.s.

*Significant differences between the two methods are in bold.n.s., not significant*.

### Tinnitus Loudness Matching

**Figure 3:**
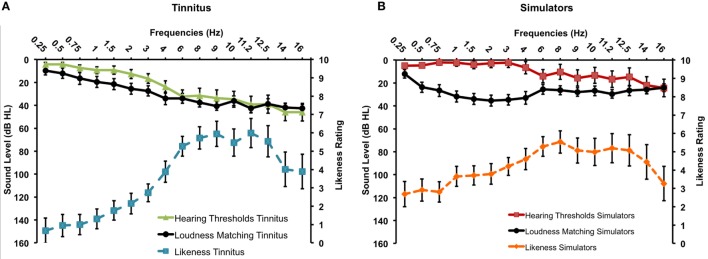
**Tinnitus spectrum (likeness ratings and loudness matches) for the Tinnitus (A) and the Simulator (B) groups (SEM)**.

On loudness ratings, there was no main effect of Methods or any interaction between Methods and Groups (all *p*s >0.10), again suggesting that both methods work similarly. However, there was a two-way interaction between Methods and Frequency, *F*(15, 420) = 2.43, *p* = 0.002: loudness matches with the Touchscreen were significantly lower for 2, 3, and 4 kHz and higher for 9 kHz (see Table 1). Overall, correlations between the two methods were again very high (mean *r* = 0.65, min: 0.26, max: 0.88). The expected group effect was significant *F*(1, 28) = 7.32, *p* = 0.011, with higher loudness matches for the Simulator compared to the Tinnitus group (mean of 17.7 and 6.1 dB SL, respectively) (see Figure 3). For the Tinnitus subgroups, there was no effect of Methods or interaction involving this factor (all *p*s >0.05). There was no main effect or interaction involving subgroups (means were: 7.97 and 4.41 dB SL for the NH and HL tinnitus, respectively).

When considering the loudness associated with the first and the second predominant pitches, the Tinnitus and Simulator groups differed significantly (see Table 2). The two Tinnitus subgroups did not differ on loudness for the two predominant frequencies.

### Testing Time

**Figure 4:**
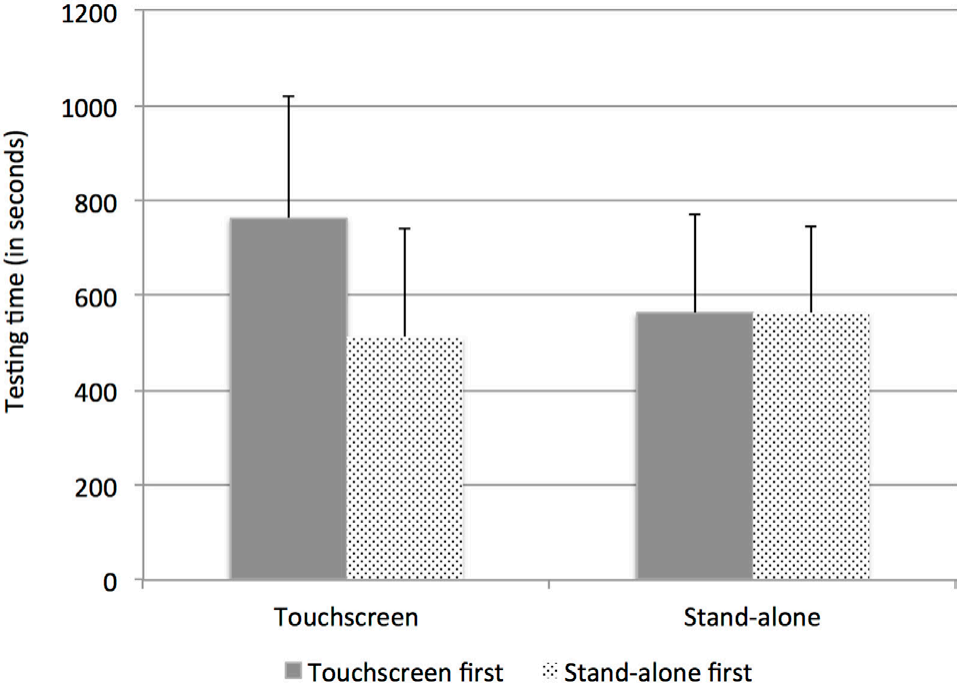
**Testing time (SD) for the two methods and the two groups according to the order of presentation (Touchscreen or Stand-alone first)**.

Figure 4 displays testing times for the two methods according to order of presentation. Analyses revealed learning effects depending on the method used, as supported by a significant interaction between Method and Order of presentation, *F*(1, 26) = 28.02, *p* < 0.001: for the Stand-alone method, the order of presentation did not matter (testing times of 9 min 22 s, n.s.) whereas when the Touchscreen method was presented first, it took significantly more time than when it was presented after the Stand-alone method (12 min 44 s vs. 8 min 32 s, *p* = 0.009). There was also a main effect of Method, with overall testing time shorter for the Stand-alone compared to the Touchscreen, *F*(1, 26) = 10,37, *p* = 0.003 (testing time Stand-alone = 10 min 46 s; range = 4 min 30 s to 20 min 01 s; testing time Touchscreen = 9 min 21 s; range: 5 min 23 s and 16 min 48 s). Finally, there was also a main effect of group, with the Simulator group being faster than the Tinnitus group, *F*(1, 26) = 7.24, *p* = 0.011 (8 min 23 s vs. 11 min 36 s for the two groups, respectively).

**Figure 5:**
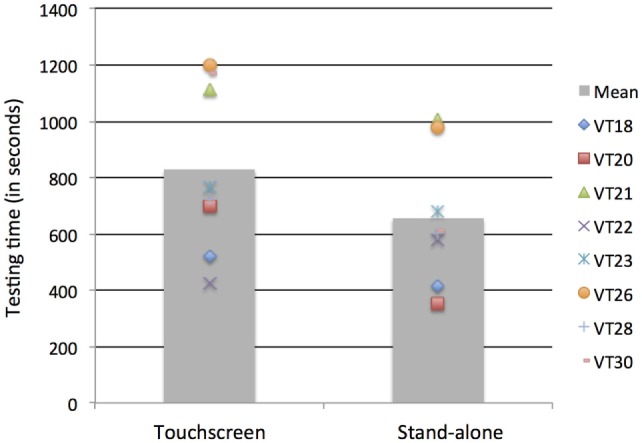
**Testing time for the subgroup Tinnitus with hearing loss using the Touchscreen or Stand-alone device**. Individual data are shown.

Analyses were also run to examine testing time differences between methods for the tinnitus subtypes (with or without HL) with the implemented audiogram. The two-way interaction between Method (Touchscreen, Stand-alone) and subgroup (without, with HL) with order of presentation as a co-variable was significant, *F*(1, 12) = 7.03, *p* = 0.021. For the subgroup without HL, testing time did not differ between methods (11 min 5 s vs. 10 min 41 s for the Touchscreen and Stand-alone, respectively, *p* = 0.75) whereas for the subgroup HL, testing time was—marginally—shorter with the Stand-alone (13 min 48 s vs. 10 min 54 s for the Touchscreen and Stand-alone, respectively, *p* = 0.052). All but one tinnitus participant with HL took less time with the Stand-alone device (see Figure 5).

### Two vs. Three Instances Presentation

The impact of presenting the two first vs. three instances of each frequency on the likeness ratings and loudness matching was also examined (see Table 3). Only two significant differences were found, one at 9 kHz for the likeness ratings (mean difference = 0.27) and one at 12.5 kHz for the loudness matches (mean difference = 3.4 dB SL). The analysis between the Tinnitus and Simulator groups was rerun on likeness and loudness matches. Similar results were obtained: for pitch matching, the frequency by groups interaction was marginally significant *F*(15, 420) = 1.62, *p* = 0.066 and *post hoc* tests revealed significant differences in ratings between the two groups for the frequencies 0.25–3 kHz, with higher ratings for the Simulators. For the loudness matching, the Frequency by Methods interaction was significant *F*(15, 420) = 2.51, *p* = 0.001, similar to the three instances results. The main group effect was also significant *F*(1, 28) = 7.14, *p* = 0.012, with Simulators having higher loudness matches (mean: 18 dB SL) than the Tinnitus participants (mean: 6.8 dB SL).

**Table 3:** **Mean differences and product-to-moment Pearson correlations between two methods instances (three vs. two) for likeness ratings and loudness matching at each frequency for merged Methods**.

	Likeness ratings				Loudness matching			
Frequency (kHz)	Mean difference	*p*-Value	*r*	*p*-Value	Mean difference (dB)	*p*-Value	*r*	*p*-Value
0.25	0.02	0.61	0.99	<0.001	−0.37	0.44	0.98	<0.001
0.5	0.05	0.41	0.99	<0.001	−0.30	0.59	0.99	<0.001
0.75	0.06	0.28	0.99	<0.001	0.002	1.0	0.99	<0.001
1	−0.07	0.12	0.99	<0.001	−0.11	0.80	0.99	<0.001
1.5	−0.09	0.45	0.96	<0.001	−0.76	0.47	0.96	<0.001
2	0.07	0.40	0.98	<0.001	1.24	0.06	0.98	<0.001
3	0.04	0.75	0.94	<0.001	−0.67	0.56	0.96	<0.001
4	0.11	0.30	0.96	<0.001	−1.73	0.25	0.94	<0.001
6	0.04	0.74	0.94	<0.001	1.03	0.17	0.98	<0.001
8	0.11	0.24	0.98	<0.001	−0.39	0.71	0.94	<0.001
9	**0.27**	**0.004**	0.98	<0.001	0.76	0.14	0.99	<0.001
10	0.08	0.40	0.98	<0.001	−1.40	0.15	0.96	<0.001
11.2	-0.01	0.97	0.99	<0.001	−0.17	0.77	0.99	<0.001
12.5	0.07	0.46	0.99	<0.001	**−3.42**	0.04	0.88	<0.001
14	0.04	0.56	0.99	<0.001	−0.67	0.10	0.99	<0.001
16	0.05	0.61	0.99	<0.001	−1.18	0.29	0.89	<0.001
Grand mean	0.05		0.98		−0.51		0.96	

*Significant mean differences between the two methods are in bold*.

### Predominant Pitch vs. Loudness as Predictors of Having Tinnitus

The logistic regression model taking tinnitus predominant pitch and loudness (in dB SL) at the tinnitus predominant pitch as predictor variables for the likelihood of having tinnitus was significant for the Touchscreen, χ^2^(2) = 6.855, *p* = 0.032. The model explained 27.2% (Nagelkerke *R*^2^) of the variance and correctly classified 70.0% of cases, with 73.3% sensitivity (i.e., correctly classifying tinnitus participants in the Tinnitus group) and 67.7% specificity (i.e., correctly classifying Simulators in the Simulator group). This model only included loudness as a significant predictor (*p* = 0.062), and not pitch (*p* = 0.90): lower loudness values were associated with greater likelihood of having tinnitus. A very similar pattern was found for the Stand-alone, χ^2^(2) = 7.261, *p* = 0.026. The model explained 28.7% (Nagelkerke *R*^2^) of the variance and correctly classified 73.3% of cases, with 73.3% sensitivity and 73.3% specificity. Likewise, the model only included loudness as a significant predictor (*p* = 0.064), and not pitch (*p* = 0.48): lower loudness values were associated with greater likelihood of having tinnitus. Running the same analyses taking only the first two instances rather than three yielded basically the same pattern of results.

## Discussion

The two methods examined here performed very similarly for participants with tinnitus as well as for participants simulating having tinnitus. Indeed, both methods produced replicable tinnitus spectrum, with similar likeness frequency ratings and very few differences in loudness matches. Both methods had highly correlated likeness ratings and loudness matches. Our study confirms that tinnitus spectrum, including frequency and loudness, can be quickly, yet robustly measured in the laboratory as well as in the clinic. Time efficiency is a major issue for clinicians and much research efforts are dedicated to the development of automated tests that are reliable and can be run while recruiting minimal clinician’s participation (11–14). Studies have endeavored to reduce test time in almost every domain of audiology including electrophysiology (13), conventional audiometry (12), speech audiometry (15), hearing screening (11), and ototoxicity monitoring (14). Our test fits perfectly in this movement by offering a testing time less than 12 min to get robust tinnitus data. While the participant is performing the task, clinicians’ expertise can be used for other purposes, such as writing reports, planning therapy, or consulting colleagues. Considering time efficiency, the Stand-alone device seems to be a better choice than the Touchscreen device for several reasons. First, the Stand-alone device has shown similar testing time independently of the order of presentation, which suggest that this device is so “user-friendly” and so easy to use, that pre-training is not necessary to improve time efficiency. In addition, the implementation of the audiogram of each patient reduces further the testing time and that is not dependent on learning effects such that it can be used confidently to measure therapeutic success or evolution of patients. This new feature might also be of particular interest to clinicians currently using the « conventional » pitch and loudness matching procedure with the audiometer. Indeed, the conventional method requires high skills from clinicians who need to consider, before each sound presentation, the frequency, the presentation level, and the degree of HL while focusing on the feedback provided by the patient during the tinnitus evaluation. These downfalls are potentially avoided with the proposed patient-directed methods. In addition, and despite the small number of participants tested in this study, the test offers good sensitivity and specificity (70% and above), lending itself for medicolegal purposes.

Presenting two pure tones instead of the standard three pure tones yielded essentially the same results. These results suggest that it could be possible to reduce testing time even more by using only two instances instead of three. This would mean that a tinnitus evaluation of pitch and loudness matching could be obtained in about 6–7 min instead of 9–10 min. Since the comparison between three and two instances was made from the same data, however, the current analysis should be taken cautiously: significant differences would mean that the third instance has a value that would be remote from the mean of the two first instances. A within-subject study design comparing two different testing sessions, one using three instances and the other one using two instances is needed before concluding on the validity of reducing the instances of presentation to two. Until further research is carried out, we believe that, for a test under 10 min, the safest solution for now would be to keep three instances.

As reported previously (6), Simulators rated lower frequencies as more similar to their tinnitus than Tinnitus participants, and overall they “matched” their tinnitus about 10 dB louder than the Tinnitus group. When considering the first and second predominant pitches of the tinnitus spectrum, only loudness matches differed between Tinnitus and Simulator participants.

Regarding tinnitus subgroups, both methods again produced comparable results, but given our small sample size, these results are tentative. Tinnitus participants with NH and HL differed mainly on the predominant pitch of their tinnitus, with—unsurprisingly—a lower predominant pitch for tinnitus participants with HL compared to those with NH. Combined with previous studies from other laboratories that have reported similar findings on likeness ratings (15–18), our study strengthens the evidence that tinnitus spectrum is mirroring the HL. Overall, the tinnitus spectrums of both tinnitus subgroups were almost identical with the exception of two very high frequencies: 14 and 16 kHz. Such differences have been noted previously, sometimes as a decline when looking at heterogeneous tinnitus groups with a wide range of HL (10) or as an increase in ratings when looking at tinnitus groups with NH (7). In the present study, hearing thresholds at those high frequencies were too elevated in the HL subgroup to be tested efficiently with either method. Considering that both subgroups (NH or HL) displayed very similar ratings for all frequencies with the exception of those two, it can be presumed that they are probably part of the tinnitus spectrum for the HL group as well, but it was not possible to assess them successfully. These results also suggest that simulators are different in their low-frequency likeness ratings from both tinnitus subgroups, either with or without HL. In addition, our study brings new information about tinnitus subgroups, in that they did not differ from one another in their loudness ratings when expressed in decibel above thresholds (dB SL). Loudness matching, an original asset of our methods compared to previous studies, is particularly important given loudness—not frequency—is the most important predictor of the presence of tinnitus. Therefore, our findings suggest that the effect of recruitment on tinnitus loudness matching did not affect significantly the results obtained by the HL subgroups. Yet, the mean loudness matching for the NH group was slightly higher (~8 dB SL) than for the HL group (~4 dB SL), which could be attributed to loudness recruitment. Most importantly, both loudness measures were lower than the Simulator group (~18 dB SL), yielding again confidence that the methods can be used with tinnitus patients displaying different types of HL. From the current data, if loudness recruitment does have an effect on tinnitus loudness matching, this aspect seems to be clinically irrelevant. Further studies with larger groups should help answering this question.

## Author Contributions

SH and PF designed the study and analyzed the data. SH wrote the manuscript and both authors finalized and approved the manuscript.

## Conflict of Interest Statement

The authors declare that the research was conducted in the absence of any commercial or financial relationships that could be construed as a potential conflict of interest.
